# Equity, acceptability and feasibility of using polyunsaturated fatty acids in children and adolescents with autism spectrum disorder: a rapid systematic review

**DOI:** 10.1186/s12955-020-01354-8

**Published:** 2020-04-16

**Authors:** Gian Loreto D’Alò, Franco De Crescenzo, Silvia Minozzi, Gian Paolo Morgano, Zuzana Mitrova, Maria Luisa Scattoni, Laura Amato, Marina Davoli, Holger J. Schünemann, on behalf of the ISACA guideline working group, Franco Nardocci, Raffaella Tancredi, Angelo Massagli, Giovanni Valeri, Corrado Cappa, Serafino Buono, Giuseppe M. Arduino, Alessandro Zuddas, Laura Reali, Massimo Molteni, Claudia Felici, Concetta Cordò, Lorella Venturini, Cristina Bellosio, Emanuela Di Tommaso, Sandra Biasci, Clelia M. Duff

**Affiliations:** 1 Department of Epidemiology, Lazio Regional Health Service, Via Cristoforo Colombo, 112, 00154 Rome, Italy; 2 School of Hygiene and Preventive Medicine, University of Rome Tor Vergata, Rome, Italy; 3 Department of Psychiatry, University of Oxford, Oxford, UK; 4 Pediatric University Hospital-Department (DPUO), Bambino Gesù Children’s Hospital, Rome, Italy; 5 Department of Health Research Methods, Evidence and Impact (formerly Clinical Epidemiology and Biostatistics, McMaster GRADE Centre, Michael G DeGroote Cochrane Canada Centre, McMaster University, Hamilton, Canada; 6 Research Coordination and Support Service, Istituto Superiore di Sanità, Rome, Italy; 7 Department of Medicine, McMaster University, Hamilton, Canada

**Keywords:** Polyunsaturated fatty acids, Autism spectrum disorder, Cost-analysis, Treatment adherence and compliance, Clinical practice guidelines

## Abstract

**Introduction:**

Some recent randomized controlled trials (RCTs) assessed the efficacy and safety of polyunsaturated fatty acids (PUFAs) for the treatment of autism spectrum disorder (ASD). To optimally inform the Italian guideline for the management of ASD in children and adolescents, we reviewed the impact on equity, acceptability and feasibility for developing a pilot recommendation for PUFAs.

**Methods:**

We performed a rapid systematic review of observational and experimental studies on PUFAs for children and adolescents with ASD, extracting data on resources required, equity, acceptability, and feasibility of PUFAs. We followed the framework provided by the grading of recommendations assessment, development and evaluation (GRADE) methodology, and we assessed risk of bias and methodological quality of included studies. Results were synthesized both narratively and quantitatively to address clinically relevant questions on equity, acceptability, and feasibility.

**Results:**

We found 14 papers related to equity. PUFAs did not seem to impact equity importantly. We did not find variation in effectiveness across subgroups and in a base case scenario, the cost of a 12 weeks cycle of therapy with 1.155 g/day of PUFAs was €65.51 euro.

The acceptability of PUFAs was evaluated in 17 studies, 9 of which were RCTs. PUFAs were widely used among children and adolescents with ASD (18 to 51%), and 50% of parents considered nutritional supplementation as useful. Difficulty in swallowing capsules and bad taste were identified as possible causes of poor compliance, but treatment adherence, when measured in included RCTs, was judged to be good to excellent. Discontinuation due to any cause for PUFAs could not differ from placebo (low certainty of evidence).

The feasibility of using PUFAs was assessed in 12 studies. PUFAs were probably sustainable, and no particular critical issue emerged from the feasibility assessment. However, the evidence appeared scarce and indirect.

**Conclusions:**

We found the administration of PUFAs in children and adolescents with ASD to be potentially equitable, acceptable and feasible. These results are limited by the limited number and quality of retrieved documents, and need to be viewed in light of efficacy and safety data to formulate clinical recommendations.

## Introduction

Children and adolescents with autism spectrum disorder (ASD) suffer from a broad set of early social communication deficits and repetitive sensory–motor behaviors. This symptomatology, that causes reduced functioning, regardless of intellectual ability, is associated with a strong genetic component as well as other causes [1].

In Italy, one of every 77 children (1.2%) in the age range between 7 and 9 years suffer from ASD [2] . In other European countries the prevalence ranges from 0.63% in Denmark and Sweden, to 1.16% in the United Kingdom. In the United States, the prevalence has been growing from 0.67% in 2000 to 1.69% in 2014, and studies estimate the prevalence in the world to be between 1 and 2% [3].

The costs of ASD are enormous for both families and society. A recent review in the United States and the United Kingdom has estimated a total lifelong cost of about 2 million euros to support a child with ASD and intellectual disability and about 1.2 million euros to support a child with ASD in the absence of intellectual disability [4], with homogeneous costs between the two countries and distributed mainly in education and in loss of parental work. In the United Kingdom, ASD is the condition with the highest socio-health costs, greater than dementia, greater than tumors, and greater than cardiovascular diseases and strokes combined [4]. Children and adolescents suffering from ASD need support from health and care services, doctors, pharmacies and hospitals all their lives. However, too many children with ASD wait far too long before obtaining a diagnosis through the National Health Service and before obtaining the care and support they need [5]. In addition, many individuals with ASD continue to have significantly worse physical and mental health than the general public and are at greater risk of dying early [6]. Treatment of ASD has been declared as fundamental for the Italian health care system, and public services are requested to model care around the needs of people [7].

While the body of evidence supporting the efficacy of psychosocial interventions is large, these treatments have limited accessibility, due to their high cost and the intense labor and parents’ involvement needed [8], so that parents usually report greater adherence to medication [9, 10]. ASD is frequently associated with psychiatric comorbidities requiring treatment [11] but, to date, only risperidone and aripiprazole have been approved by the Food and Drug Administration (FDA) for the treatment of irritability in ASD, with no medications approved by the European Medicines Agency (EMA), and no pharmacological treatment proved to be effective in treating core symptoms. Concerns about adverse events often shift parents to prefer complementary and alternative medicines (CAMs) [8, 12].

A number of health supplement foods are on the market, and products for children have also increased [13]. The potential beneficial effect of the administration of specific nutrition supplements (e.g. fatty acids, vitamins, minerals) has been explored in several studies, and the use of CAMs in ASD is on the rise [8, 14], with the majority of parents of children with ASD reporting the use of some type of dietary intervention [14].

Polyunsaturated fatty acids (PUFAs) are fatty acids containing at least two carbon-carbon double bonds in their carboxylic chain, and classified into omega-3, omega-6 and omega-9 [15]. Their role in the prevention of cardiovascular diseases has been widely studied [15], while the interest for the use of PUFAs, especially considering eicosapentaenoic acid (EPA) and docosahexaenoic acid (DHA), two omega-3 fatty acids, in the treatment of psychiatric diseases has been rising in recent years [16, 17].

Feasibility and adherence to PUFAs administration are not clear. Children often display difficulties with swallowing tablet-form and liquid-form medications [18], and these difficulties are increased among individuals with developmental disabilities.

## Methods

This systematic review was performed to support the development of the Italian National Institute of Health (in Italian: Istituto Superiore di Sanità - ISS) evidence-based guidelines for the diagnosis and management of children and adolescents with ASD. In accordance with the ISS methodological manual for clinical practice guidelines (GL) development [19], the ISS guideline group formulated 15 questions for developing evidence-based health recommendations [20–22]. The multidisciplinary panel, including caregivers of children/adolescents with ASD, agreed to follow Grading of Recommendations Assessment, Development and Evaluation (GRADE) methodology and the Evidence to Decision (EtD) framework to support their decisions. The use of the EtD framework in general, and the evaluation of its domains relating to equity, acceptability and feasibility in particular, requires the panel to be familiar with the tool [23]. The Evidence Review Team together with the ISS principal investigator and the GL chairs decided the following clinically relevant questions on the use of PUFAs, with the aim of training the panel members in structuring discussion, saving time, ensuring systematicity in the process of formulating the recommendations [24]. We reported elsewere the results of our systematic review on the efficacy and safety of PUFAs in children and adolescents with ASD [25]. Here we report the results of the systematic review on equity, acceptability and feasibility, as contextual factors that influence recommendations developing using the GRADE EtD framework [23].

### The questions

- What would be the impact of PUFAs on health equity?
- Are PUFAs acceptable to key stakeholders?
- Are PUFAs feasible to implement?

### Population

Children and adolescents aged 0–18 years, of both genders, with a primary diagnosis of autism spectrum disorder.

### Intervention

Any type and any dose of PUFAs.

### Outcomes

Equity, defined as the presence of plausible reasons for anticipating differences in the relative effectiveness of the intervention for disadvantaged subgroups or different baseline conditions across disadvantaged subgroups that affect the absolute effectiveness of the intervention or the importance of the problem [15].

Acceptability, defined as the likelihood for the key stakeholders to accept or agree with: the distribution of the benefits, harms and costs; the costs or undesirable effects in the short term for desirable effects (benefits) in the future; the values attached to the desirable or undesirable effect [15]; discontinuation due to any cause, as measured in randomized controlled trials (RCT) and compared vs placebo, was also included as a proxy of acceptability. We produced a summary of findings table based on the methodology developed from the GRADE Working Group [26–30] assigning four levels of certainty in the evidence (high, moderate, low, very low) based on different ratings on study limitations, indirectness, inconsistency, imprecision of effect estimates, and risk of publication bias. We accompanied the results with a narrative statement, as advised by Santesso et al. (2019) [31].

Feasibility, defined as sustainability of the intervention and the capability to address potential barriers to using it [26].

### Types of studies included

We initially searched for systematic reviews and overviews of the literature on acceptability, equity and feasibility.

Because we did not find any existing systematic review focusing on the specific domains mentioned above (i.e. acceptability, equity, feasibility), we searched for primary studies. For each considered outcome we included both observational and experimental studies on PUFAs. The literature search was conducted up to October 2018. No language filters were applied.

### Literature search

1) For equity, we searched national cost data on national databases (such as Farmadati- https://www.farmadati.it/) [32] on costing and resources of PUFAs. We ran the search strategy reported in Additional File 1 for systematic reviews and primary studies of both experimental and observational studies including data on equity (see Additional File 1). In addition, we performed an unstructured search on PubMed and Google Scholar in order to find published studies and grey literature on socioeconomic and cultural aspect that could impact equity.

2) For acceptability, we searched Medline through PubMed for systematic reviews and primary studies of both experimental and observational studies. The full search strategy is available in the Additional file 1. References of the included studies were thoroughly inspected to look for other studies potentially relevant for inclusion.

3) For feasibility, we searched PubMed for systematic reviews and primary studies dealing with feasibility of treatment with PUFA through the search strategy presented in Additional file 1. We also performed on PubMed unstructured searches.

### Study selection and data extraction

Two reviewers (GLD, FDC) independently screened titles and abstracts of all publications that were obtained by the search strategy. The same authors independently assessed the full text of potentially relevant studies for inclusion. Disagreement was resolved by a consensus meeting or by a third reviewer (SM).

Two reviewers (GLD, FDC) independently extracted data

#### Equity

Resources required may have an impact on equity. We reviewed data on resources needed to implement the treatment with PUFAs. In detail, we extracted the prices of the supplements, the dosage of PUFAs administered daily in RCTs, and the length of treatment according to RCTs. We also reviewed non-randomized studies that analyzed other socio-economic and cultural aspect that could have an impact on equity. We extracted data on the influence of several factors (i.e age, disability, sexual orientation, time-dependent situations, relationships, place of residence, race/ethnicity/culture, language, occupation, gender/sex, religion, education, socioeconomic status, or social capital) on the absolute effectiveness of PUFAs or the importance of the problem [33, 34]. We extracted both quantitative and qualitative data. Finally, we extracted data on equity from RCTs on PUFAs in children and adolescents with ASD.

#### Acceptability

Systematic reviews and primary studies were inspected and relevant data described narratively. We also evaluated the acceptability in term of discontinuation due to any cause from RCTs.

#### Feasibility

We inspected the background and discussion sections of the retrieved RCTs on efficacy and non-randomized studies to look for data, consideration and additional references on barriers to the implementation of therapy and sustainability of the intervention.

### Data analysis and synthesis

Different approaches were used to synthetize data for the different outcomes considered in our systematic review, in detail:

For equity, we performed a quantitative synthesis of data on resources required, in order to obtain the estimated cost of a day of therapy and of a treatment cycle; data on diverse anticipated effectiveness across subgroups were synthetized narratively. For acceptability, we synthetized narratively data on actual use and adherence extracted from both RCTs and non-randomized studies; discontinuation due to any cause was synthetized through calculating the Risk Ratio and its confidence interval.

For feasibility, we summarized narratively data retrieved from systematic reviews and primary studies regarding feasibility. Moreover, we included equity and acceptability features (i.e. resources required, adherence and discontinuation due to any cause) in the qualitative synthesis of evidence on feasibility.

### Quality assessment

For RCTs, we used the Cochrane tool for risk of bias assessment [35]. The following domains were assessed: sequence generation; allocation concealment; blinding of participants, providers and outcome assessors; incomplete outcome data; selective reporting; other bias (e.g. funding source, baseline imbalance, interventions poorly delivered). A ‘Risk of bias’ table was created for the included studies, which indicates the study’s performance in each of the above-mentioned domains. For each domain, a judgment was assigned in terms of low, high, or unclear risk of bias.

We used the Risk Of Bias In Non-randomized Studies – of Interventions (ROBINS-I) tool [36] for assessing risk of bias of Controlled Clinical Trials and before-after studies and a modified version of NOS [37] for cross-sectional studies. The following domains were assessed for cross-sectional studies: representativeness of the sample; sample size; non-respondents; ascertainment of the exposure (risk factor); comparability of outcome groups; assessment of the outcome; statistical test. A NOS table was created for the included studies, which indicates the study’s performance in each of these domains.

We used the Quality of Health Economic Studies (QHES) instrument for rating the quality of included cost-analysis studies [38]; quality assessments were performed by two independent reviewers (GLD, FDC), with final scoring based on consensus to ensure a reliable quality score [39].

## Results

### Studies retrieved through the search strategy for RCTs

We retrieved 786 citations from database searching, 33 records from trial registers and one additional document from other sources. After removing 228 duplicates, we excluded 554 citations from title or abstract. Of the remaining 38 full text articles, 15 were excluded for various reasons (see Additional file 2).

**Fig. 1:**
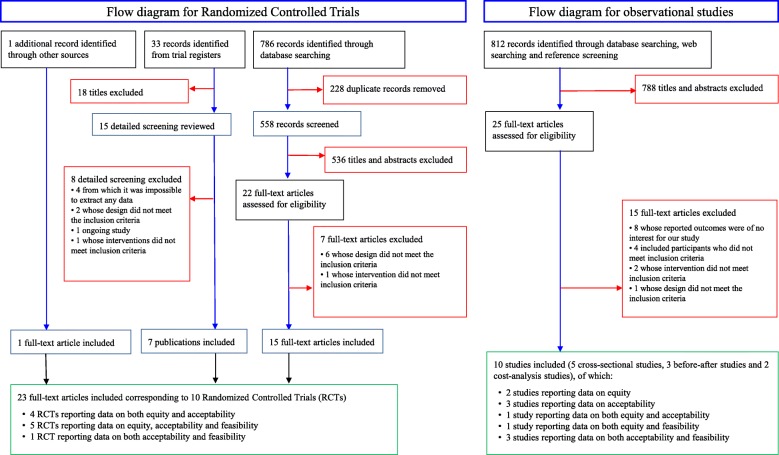
Flow diagram

Finally, we identified 23 full text articles corresponding to 10 RCTs [40–49] through the search strategy for experimental studies (see also Additional file 2 and Fig. 1).

### Non-randomized studies retrieved through the search strategy

We retrieved 809 citations from database searching and 3 studies from unstructured web searching. We excluded 788 documents from title and abstract, and after excluding 15 full text articles for various reasons (see Additional file 2) we finally included 10 non-randomized studies reporting data on equity, acceptability or feasibility [9, 41–58] (see Fig. 1).

### Equity

#### Resources required

We found one article [50] and one thesis [51] reporting data about the cost of PUFAs in the world. To obtain data on the dosage in the population of interest we have used the data contained in the RCTs [40–48] (see Fig. 1). The price of the drug in Italy has been obtained by selecting all the drugs currently on the market and Class A prescription drugs according to AIFA notes 13 and 94. The data relating to the number of capsules per package, the dosage of the single capsule and the price of the package were obtained from Italian Databases of Medicine, Parapharmaceutical and Medical Device [32].

Watters et al. [50] identified supplements as the cheapest way, immediately after fish liver oil, to take a high dosage (≥500 mg/day) of PUFAs such as DHA and EPA. The estimated cost of the equivalent of a tablet containing 1000 mg of EPA plus DHA was $0.88 ± $0.16 in the USA [33]. Similarly, according to another USA study, which considered a wider range of marketed PUFAs’ supplements, the estimated cost of the equivalent of a tablet containing 1000 mg EPA plus DHA was $0.70 ± $1.11 [51]. The quality of the included cost-analysis studies, as rated through the Quality of Health Economic Studies (QHES) instrument, was of 67 and 61 out of the 100 potential points for Watter et al. [50] and Press [51] study, respectively. Detailed ratings for each item are reported in Additional file 3. Italian data were consistent with these findings, since the median price per 1000 mg of net product was €0.68, ranging from a minimum of €0.65 to a maximum of €0.83 [32].

When considering RCTs [40–48], the median daily dose of PUFAs administered for the treatment of autism symptoms was 1155 mg/day, ranging from 200 mg/day to 1540 mg/day. Taking into account the above data regarding the dosages used in RCTs and the estimated prices of drugs, the cost of a day of therapy should range from €0.13 to €1.28, with a median value of €0.78. In RCTs, treatment cycles duration varies from 6 to 52 weeks, with a median duration of 12 weeks. Considering this variability, together with the price and dosage variability, we calculated that, in a base case scenario in which 1155 mg/day of PUFAs were administered for 12 weeks at a cost of €0.68/1000 mg, the cost of a cycle of therapy would be €65.51 euro. The complete sensitivity analysis, with the minimum and maximum expenditure scenarios, is shown in Table 1.

**Table 1:** Sensitivity analysis of the costs for PUFAs administration

	Estimated costs for a therapeutic cycle (€)		
	Lower scenario	Base case scenario	Upper scenario
Short therapeutic cycle (6 weeks)	5,5	32,8	53,7
Intermediate therapeutic cycle (12 weeks)	10,9	65,5	107,4
Long therapeutic cycle (52 weeks)	47,5	284,7	466,5

Since PUFAs are not indicated for the therapy of ASD symptomatology, are not reimbursed by the Italian National Health Service (Sistema Sanitario Nazionale - SSN), and are fully borne by the family of the patient.

#### Diverse anticipated effectiveness across subgroups

We found two cross-sectional studies [9, 58] reporting data on equity for children and adolescents with ASD. Quality for these studies was rated as high (7/10 stars) (Additional file 4).

According to Salomone et al. [58], parents’ education influences the choice to undertake an alternative or complementary therapy, such as that with PUFAs, while Hock et al. [9] survey showed that the perceived burden of the therapy in terms of time, money and energy would affect adherence to the treatment. However, the authors argue that taking medication or supplements should have a low impact on the commitment of family caregivers, as it is a relatively concrete and limited task for parents to perform.

### Acceptability

#### Actual use and adherence predictive factors

We found four cross-sectional studies [9, 52–54], three before-after studies [55–57] and two RCTs [45, 49] reporting data on actual use and adherence predictive factors for children and adolescents with ASD (see Fig. 1). Although the studies were conducted and analyzed appropriately for cross-sectional studies, one study [52] was judged of poor quality; risk of bias for before-after studies was judged serious [56, 57] to critical [55] for the assessment of adherence (Additional file 5); Voigt et al. [45] was judged as high risk of bias for incomplete outcome data, while we felt risk of bias was unclear for lack of blinding, incomplete outcome data and other bias; Keim et al. [49] was judged at unclear risk for other bias, due to uncertainties in the diagnostic criteria (Additional file 6) .

A survey aimed at exploring parental perspectives on use and benefits of CAM in children with ASD showed that the use of nutritional supplement in this population seems to be quite widespread, with 55% using at least one nutritional supplement; PUFAs were used by 18% of the individuals [52]. According to another survey [53], however, 51% of children with ASD used PUFAs in the USA. Nutritional supplementation was considered useful by 50% of parents of individuals with autism spectrum disorder (ASD).

According to a survey that assessed the parent-reported adherence to ASD treatments, the average adherence to treatment with an alternative approach, such as the use of PUFAs, was significantly lower than adherence to drug therapy or evolutionary therapy, while it was comparable to adherence to behavioral therapy. According to this study, an important predictive factor of adherence was the perceived burden of the therapy on the family in terms of time, energy, and money [12].

Difficulty with swallowing the capsules may be another challenge in administering PUFAs supplements to children with ASD [54]. An alternative would be to provide PUFAs in a liquid formulation, more suitable for children, especially if they are pre-schoolers. However, the same liquid formulation could potentially reduce treatment compliance, due to the sensory characteristics that typically characterize fish oil supplements [49, 54, 56]. In one RCT [45], to promote adherence to treatment, it was decided to opt for a capsule containing a lower dosage of PUFAs (200 mg/day); the authors avoided administration of higher or frequent dosages because a single daily dose is already challenging in the autistic population.

#### Observed adherence and discontinuation (concurrent and retrospective acceptability)

We found results reported on adherence and discontinuation in ten RCTs [40–49] and in two before-after studies [55, 57] (see Fig. 1). We evaluated the risk of bias for the included RCTs (Additional file 6). Three studies [41, 42, 44] were judged as low risk of bias for all the considered domains. Only one study [47] was judged as high risk of bias for issues with random sequence generation and for lack of blinding, while another study [45] was at high risk of bias for incomplete outcome data. One study [40] was at unclear risk of bias in four domains (random sequence generation, allocation concealment, blinding, and other sources of bias), while the remaining two studies [46, 48] were at unclear risk of bias for incomplete outcome data. One study [49] was judged to be at unclear risk of bias for uncertainties in diagnostic criteria for ASD. About the before-after studies, risk of bias ranged from severe [57] to critical [55] (Additional file 5).

Discontinuation due to any reason for PUFAs does not differ from that of placebo in clinical studies (RR 1.01, 95%CI 0.66 to 1.54; low certainty of evidence), and was studied in 7 studies [40–45, 48] with a total population of 315 children and adolescents with ASD (Table 2). We included in these analyses an insufficient number of studies to perform a meaningful presentation of publication bias through funnel plots [59] but judged publication bias not to be of concern.

**Table 2:** Discontinuation due to any reasons in RCTs comparing PUFAs vs placebo: summary of findings

Should polyunsaturated fatty acids versus placebo be used for the treatment of children and adolescents with autism spectrum disorder?						
**Patient or population:** children and adolescents with autism spectrum disorder **Setting:** outpatients **Intervention:** polyunsaturated fatty acids (PUFAs) **Comparison:** placebo						
Outcomes	Anticipated absolute effects^*^ (95% CI)		Relative effect(95% CI)	№ of participants (studies)	Certainty of the evidence(GRADE)	Comments
	**Risk with placebo**	**Risk with PUFAs**				
Discontinuation due to any cause	213 per 1.000	**226 per 1.000**(119 to 432)	**RR 1.06**(0.56 to 2.03)	315 (7 RCTs) [20–25, 27]	⨁⨁◯◯ LOW ^a^	Polyunsaturated fatty acids could have no effect on the risk of discontinuation due to any cause.

*The risk in the intervention group (and its 95% confidence interval) is based on the assumed risk in the comparison group and the **relative effect** of the intervention (and its 95% CI).

**CI:** Confidence interval; **RR:** Risk ratio; **SMD:** Standardised mean difference

**GRADE Working Group grades of evidence**

**High certainty:** We are very confident that the true effect lies close to that of the estimate of the effect

**Moderate certainty:** We are moderately confident in the effect estimate: The true effect is likely to be close to the estimate of the effect, but there is a possibility that it is substantially different

**Low certainty:** Our confidence in the effect estimate is limited: The true effect may be substantially different from the estimate of the effect

**Very low certainty:** We have very little confidence in the effect estimate: The true effect is likely to be substantially different from the estimate of effect

^a^Downgraded of two levels because optimal information size (OIS) not met and there is a wide 95%CI, which includes no effect

Several RCTs also evaluated adherence to treatment, both subjectively and objectively (i.e. self-report, pills count, laboratory analyses). In these studies, adherence was considered good to excellent [41, 44, 45, 47–49]. In detail, Mazahery et al. [48] reported, in the patients randomized to PUFAs or to PUFAs plus vitamin D, an increase in the omega-3 index of 4.4 and 4%, respectively, compared to baseline, while Voigt et al. [45] reported a significant increase in plasmatic DHA levels in all the individuals randomized to PUFAs, with a median increase of 430% in plasmatic DHA levels; Johnson et al. [47] reported that only one participant in the study (10%) did not regularly take the medication, while in the study by Bent et al. [41], treatment compliance was judged to be perfect or almost perfect in 69% of patients randomized to PUFAs, compared to 75% of patients randomized to placebo. Very high adherence rates (97%) are reported in both intervention and placebo arms by [44].

An observational study [57] seems to confirm the good acceptability of PUFAs as measured by treatment discontinuation with 8 out of 9 patients completing 12-week treatment. Finally, among the evidence from observational studies, Belmaker et al. [55] advised the administration of PUFAs to 250 children and adolescents with ASD in the context of a pre-school psychiatric clinic for ASD in Israel [55]. About two-thirds of the children agreed to take the medication. Half of those who agreed to take PUFAs then stopped taking it because no improvement was observed or because of the bad taste. No barriers other than bad taste were found [55]. However, risk of bias in these before-after studies varied from serious [57] to critical [55].

### Feasibility

We found four cross-sectional studies [9, 52, 54, 58], one before-after study [56], and one RCT [45] reporting data on feasibility of the implementation of PUFAs therapy in children and adolescents with ASD. We also considered adherence data from six RCTs [41, 44, 45, 47–49] as a proxy for evaluating feasibility (see Fig. 1). For risk of bias assessment in RCTs, please see Acceptability section and Additional file 6. Three surveys [9, 54, 58] were considered of good quality, with scores ranging from seven to eight of ten stars (see also Additional file 4). The before-after study by Ooi et al. [56] was considered at serious risk of bias (Additional file 5).

Huang et al. [52] showed in their survey that doctors are not perceived by parents of people with ASD as sufficiently well informed about alternative therapies for ASD, including PUFAs. Salomone et al. [58] suggested that parents would choose to undertake alternative or complementary therapies based on the advice of nurses, nutritionists and alternative physicians. The authors, therefore, argue that physicians should be able to discuss the efficacy and possible risks of alternative or complementary treatments, including PUFAs, with the parents of subjects with ASD.

Among the barriers to the implementation of therapy with PUFAs we believe that the lack of adherence is a result of the difficulties in convincing children or adolescents with ASD to take the medication (difficulty in swallowing the capsule, unpleasant sensory characteristics of the product) [45, 49, 54, 56], and of the personal opinion of the parent about the effectiveness and usefulness of the treatment [8]. Nevertheless, in the RCTs that we considered as addressing acceptability, adherence to therapy was generally good or excellent [41, 44, 45, 47–49].

## Discussion

We performed a systematic review to evaluate factors related to a potential treatment of ASD that are increasingly considered critical for decision making in general and for guideline recommendations in particular: equity, acceptability and feasibility. We found a number of studies that provided results which were used by the guideline panel to formulate recommendations about PUFA for ASD. Despite this, some uncertainties remain in the three domains related to equity, acceptability and feasibility. About equity, uncertainty in the cost assessment results from the lack of data on the specific cost for children and adolescents with ASD; there are also uncertainties regarding the cost of tablets with EPA dosages plus DHA below 500 mg/day. In fact, among the class A drugs for the secondary prevention of cardiovascular disease, there was no tablet with a dosage of less than 500 mg of PUFA [32], while for the minimum expenditure scenario a dosage of 200 mg/day was considered [45], a formulation for which no cost data were available. The costs of the minimum expenditure scenarios could therefore be slightly higher, given the relative weight of the fixed costs of packaging and distribution. Regarding acceptability, the information we collected on the use of PUFAs in children and adolescents with ASD, as well as those on adherence to CAM therapies, which could be considered as prospective acceptability, referred mostly to questionnaires administered during non-interventional observational studies. Information on concurrent acceptability, as well as retrospective acceptability, was not systematically collected in the trials, but derived from spontaneous parental reporting, investigator observations, or inferred by proxies like discontinuation and adherence. We chose to include discontinuation due to any cause as a proxy of acceptability, but reasons why patients withdraw their participation may or may not be associated with acceptability [60]. About feasibility, we did not find any study focusing on feasibility of implementing PUFAs therapy in children and adolescents with ASD, nor in other populations. For this reason, we mainly relied on the results collected for the equity and acceptability domains as indirect evidence of feasibility.

### Limitations

The main study limitation is related to the nature of retrieved literature, as the assessment of feasibility, acceptability, equity was limited due to the scarce number of publications, and poor design quality: also due to the nature of the rapid systematic review [61], our findings are based on the relatively small number of studies retrieved through our search strategy and meeting our inclusion criteria. We cannot rule out that we would have found relevant papers by running a search strategy focusing on equity, acceptability, and feasibility on databases other than Medline, and by adding additional keywords (i.e. other omega-3 and omega-6 molecules) to the search string, and conclusions may be subject to change and/or revision once a more comprehensive systematic review has been completed. However, in an attempt to limit publication bias, we carried out a focused search of gray literature to corroborate our findings.

### Strength

We addressed an emerging field in systematic review research, the conduct of evidence synthesis of contextual evidence that influences recommendations. This evidence has in the past been used only anecdotally and guideline developers often failed to systematically look for evidence.

We believe that this early example of systematically attempting to summarize this evidence provides a strength to our work.

## Conclusions

The resources required for the implementation of PUFAs therapy in children and adolescents with ASD are likely to be limited, and PUFAs are not expected to have any negative or positive impacts on equity. Patient and caregiver acceptability of treatment could be good in terms of both adherence and discontinuation (low certainty of evidence). Difficulties in pill-swallowing could be lessened by behavioral training [62]. Physician acceptability may vary based on personal knowledge of alternative therapies for ASD [52, 58], and this may also adversely affect feasibility. Overall, the implementation of PUFAs is likely to be feasible: in fact, PUFAs are already administered to a significant portion of individuals with ASD [52, 53]. The decision to allocate resources in the administration of PUFAs to children and adolescents with ASD needs to be based on weighing the costs and difficulties of implementation, whose estimates are still based on scarce evidence, with the available evidence of effectiveness and safety, which have not been yet demonstrated [25].

## Supplementary information

**Additional file 1.** Search strategy.

**Additional file 2.** References for included and excluded studies.

**Additional file 3.** Quality of Health Economic Studies (QHES) instrument rating for included cost-analysis studies.

**Additional file 4.** Newcastle-Ottawa Scale for included cross-sectional studies.

**Additional file 5.** ROBINS-I for included before-after studies.

**Additional file 6.** Risk of bias summary for included randomized controlled trials.

## Data Availability

All data supporting our findings is contained within the manuscript and the additional files. The authors can be contacted at f.decrescenzo@deplazio.it (FDC) for further clarification, if required.

## Abbreviations

ASD: Autism Spectrum Disorder
CAM: Complementary and Alternative Medicines
DHA: docosahexaenoic acid
EPA: Eicosapentaenoic acid
EtD: Evidence to Decision
GRADE: Grading of Recommendations Assessment, Development and Evaluation
PUFA: Polyunsaturated fatty acid
RCT: Randomized Controlled Trial
