# Supplementary material for: Equity, acceptability and feasibility of using polyunsaturated fatty acids in children and adolescents with autism spectrum disorder: a rapid systematic review
Source: Health Qual Life Outcomes. 2020 Apr 16;18:101. doi: 10.1186/s12955-020-01354-8 (PMC7164335; doi:10.1186/s12955-020-01354-8)
Supplement: Supplementary file 1 — Additional file 1. Search strategy. [file 12955_2020_1354_MOESM1_ESM.docx]

**Additional file 1.**

# Search Strategy for experimental studies.

# 1. CENTRAL search strategy

**CENTRAL (via onlinelibrary.wiley.com)**

1. MeSH descriptor: [Fatty Acids] this term only
2. MeSH descriptor: [Fatty Acids, Omega-3] explode all trees
3. MeSH descriptor: [Fatty Acids, Omega-6] explode all trees
4. (omega NEXT 3 or omega NEXT 6)
5. ((eicosa* or icosa* or docosahex* or alpha-linol*) NEXT acid*)
6. poly NEXT unsaturat* NEXT fatty NEXT acid*
7. polyunsaturat* NEXT fatty NEXT acid*
8. essential NEXT fatty NEXT acid*
9. PUFA*
10. MeSH descriptor: [Fish Oils] this term only
11. MeSH descriptor: [Linseed Oil] this term only
12. (EFA or EPA or MaxEPA or DHA or ALA)
13. (oil* NEAR/3 (fish* or flax or linseed))
14. #1 or #2 or #3 or #4 or #5 or #6 or #7 or #8 or #9 or #10 or #11 or #12 or #13
15. MeSH descriptor: [Child Development Disorders, Pervasive] explode all trees
16. asperger*
17. autis* or ASD or ASDs
18. kanner*
19. rett*
20. childhood near/3 schizophren*
21. (pervasiv* NEXT development* NEXT disorder*) OR PDD or PDDs
22. MeSH descriptor: [Social Behavior Disorders] this term only
23. MeSH descriptor: [Child Behavior Disorders] this term only
24. MeSH descriptor: [Communication Disorders] this term only
25. (behav* or communicat*) NEXT (disorder* or impair*)
26. #15 or #16 or #17 or #18 or #19 or #20 or #21 or #22 or #23 #24 or #25
27. #14 and #26 in Trials

# 2. MEDLINE search strategy

**MEDLINE (Ovid MEDLINE® Epub Ahead of Print, In-Process & Other Non-Indexed Citations, Ovid MEDLINE® Daily and Ovid MEDLINE®) 1946 to 2018 October 26.**

1. exp child development disorders, pervasive/
2. Developmental Disabilities/
3. pervasive development$ disorder$.tw.
4. (pervasive adj3 child$).tw.
5. (PDD or PDDs or ASD or ASDs).tw.
6. autis$.tw.
7. asperger$.tw.
8. kanner$.tw.
9. 1 or 2 or 3 or 4 or 5 or 6 or 7 or 8
10. Fatty Acids/
11. fatty acids, unsaturated/
12. exp Fatty Acids, Omega-3/
13. exp Fatty Acids, Omega-6/
14. alpha-Linolenic Acid/
15. Docosahexaenoic Acids/
16. Eicosapentaenoic Acid\
17. (omega 3 or omega 6).tw.
18. polyunsaturat$ fatty acid$.tw.
19. PUFA.tw.
20. Fish Oils/
21. linseed oil/
22. (EFA or EPA or MaxEPA or DHA or ALA).tw.
23. (oil$ adj3 (fish$ or flax or linseed)).tw.
24. 10 or 11 or 12 or 13 or 14 or 15 or 16 or 17 or 18 or 19 or 20 or 21 or 22 or 23
25. 9 and 24
26. randomized controlled trial.pt.
27. controlled clinical trial.pt.
28. randomi#ed.ab.
29. placebo$.ab.
30. drug therapy.fs.
31. randomly.ab.
32. trial.ab.
33. groups.ab.
34. 26 or 27 or 28 or 29 or 30 or 31 or 32 or 33
35. exp animals/ not humans.sh.
36. 34 not 35
37. 25 and 36
38. meta-analysis.pt.
39. meta-analysis/ or systematic review/ or meta-analysis as topic/ or "meta analysis (topic)"/ or "systematic review (topic)"/ or exp technology assessment, biomedical/
40. ((systematic* adj3 (review* or overview*)) or (methodologic* adj3 (review* or overview*))).ti,ab,kf,kw.
41. ((quantitative adj3 (review* or overview* or synthes*)) or (research adj3 (integrati* or overview*))).ti,ab,kf,kw.
42. ((integrative adj3 (review* or overview*)) or (collaborative adj3 (review* or overview*)) or (pool* adj3 analy*)).ti,ab,kf,kw.
43. (data synthes* or data extraction* or data abstraction*).ti,ab,kf,kw.
44. (handsearch* or hand search*).ti,ab,kf,kw.
45. (met analy* or metanaly* or technology assessment* or HTA or HTAs or technology overview* or technology appraisal*).ti,ab,kf,kw.
46. (meta regression* or metaregression*).ti,ab,kf,kw.
47. (meta-analy* or metaanaly* or systematic review* or biomedical technology assessment* or bio-medical technology assessment*).mp,hw.
48. (medline or cochrane or pubmed or medlars or embase or cinahl).ti,ab,hw.
49. (cochrane or (health adj2 technology assessment) or evidence report).jw.
50. (comparative adj3 (efficacy or effectiveness)).ti,ab,kf,kw.
51. (outcomes research or relative effectiveness).ti,ab,kf,kw.
52. ((indirect or indirect treatment or mixed-treatment) adj comparison*).ti,ab,kf,kw.
53. 38 or 39 or 40 or 41 or 42 or 43 or 44 or 45 or 46 or 47 or 48 or 49 or 50 or 51 or 52
54. 25 and 53
55. Economics/
56. exp "Costs and Cost Analysis"/
57. Economics, Nursing/
58. Economics, Medical/
59. Economics, Pharmaceutical/
60. exp Economics, Hospital/
61. Economics, Dental/
62. exp "Fees and Charges"/
63. exp Budgets/
64. budget*.ti,ab,kf.
65. (economic* or cost or costs or costly or costing or price or prices or pricing or pharmacoeconomic* or pharmaco-economic* or expenditure or expenditures or expense or expenses or financial or finance or finances or financed).ti,kf.
66. (cost* adj2 (effective* or utilit* or benefit* or minimi* or analy* or outcome or outcomes)).ab,kf.
67. (value adj2 (money or monetary)).ti,ab,kf.
68. exp models, economic/
69. economic model*.ab,kf.
70. markov chains/
71. markov.ti,ab,kf.
72. monte carlo method/
73. monte carlo.ti,ab,kf.
74. exp Decision Theory/
75. (decision* adj2 (tree* or analy* or model*)).ti,ab,kf.
76. 55 or 56 or 57 or 58 or 59 or 60 or 61 or 62 or 63 or 64 or 65 or 66 or 67 or 68 or 69 or 70 or 71 or 72 or 73 or 74 or 75
77. 25 and 76
78. 37 or 54 or 77

# 3. EMBASE search strategy

**EMBASE (via OVID)**

**Database: Embase Classic + Embase, 1947 to 2018 October 26.**

1. fatty acid/
2. omega 3 fatty acid/ or omega 6 fatty acid/
3. docosahexaenoic acid/
4. linolenic acid/
5. icosapentaenoic acid/
6. (omega 3 or omega 6).tw.
7. ((eicosa$ or icosa$ or docosahex$ or alpha-linol$) adj acid$).tw.
8. polyunsaturat$ fatty acid$.tw.
9. poly-unsaturat$ fatty acid$.tw.
10. (fatty acid$ adj3 n-3).tw.
11. essential fatty acid$.tw.
12. PUFA.tw.
13. Fish Oils/
14. linseed oil/
15. (EFA or EPA or MaxEPA or DHA or ALA).ti,ab.
16. (oil$ adj3 (fish$ or flax or linseed)).tw.
17. 1 or 2 or 3 or 4 or 5 or 6 or 7 or 8 or 9 or 10 or 11 or 12 or 13 or 14 or 15 or 16
18. autis$.tw.
19. PDD.tw.
20. pervasive developmental disorder$.tw.
21. kanner$.tw.
22. (asperg$ not aspergill$).tw.
23. autism/ or asperger syndrome/ or infantile autism/
24. 18 or 19 or 20 or 21 or 22 or 23
25. 17 and 24
26. exp clinical trial/
27. exp crossover procedure/
28. exp double blind procedure/
29. exp controlled clinical trial/
30. (placebo or assign* or allocat* or volunteer* or random* or factorial* or crossover).ti,ab.
31. ((singl$ or doubl$ or trebl$ or tripl$) adj3 (blind$ or mask$)).tw.
32. 26 or 27 or 28 or 29 or 30 or 31
33. 25 and 32
34. meta-analysis/ or systematic review/ or meta-analysis as topic/ or "meta analysis (topic)"/ or "systematic review (topic)"/ or exp technology assessment, biomedical/
35. ((quantitative adj3 (review* or overview* or synthes*)) or (research adj3 (integrati* or overview*))).ti,ab,kw.
36. ((integrative adj3 (review* or overview*)) or (collaborative adj3 (review* or overview*)) or (pool* adj3 analy*)).ti,ab,kw.
37. (data synthes* or data extraction* or data abstraction*).tw.
38. (handsearch* or hand search*).ti,ab.
39. (met analy* or metanaly* or technology assessment* or HTA or HTAs or technology overview* or technology appraisal*).ti,ab,kw.
40. (meta regression* or metaregression*).ti,ab,kw.
41. (meta-analy* or metaanaly* or systematic review* or biomedical technology assessment* or bio-medical technology assessment*).mp,hw.
42. (medline or cochrane or pubmed or medlars or embase or cinahl).ti,ab,hw.
43. (cochrane or (health adj2 technology assessment) or evidence report).jw.
44. (comparative adj3 (efficacy or effectiveness)).ti,ab,kw.
45. (outcomes research or relative effectiveness).ti,ab,kw.
46. ((indirect or indirect treatment or mixed-treatment) adj comparison*).ti,ab,kw.
47. 34 or 35 or 36 or 37 or 38 or 39 or 40 or 41 or 42 or 43 or 44 or 45 or 46
48. 25 and 47
49. Economics/
50. Cost/
51. exp Health Economics/
52. Budget/
53. budget*.ti,ab,kw.
54. (economic* or cost or costs or costly or costing or price or prices or pricing or pharmacoeconomic* or pharmaco-economic* or expenditure or expenditures or expense or expenses or financial or finance or finances or financed).ti,kw.
55. (cost* adj2 (effective* or utilit* or benefit* or minimi* or analy* or outcome or outcomes)).ab,kw.
56. (value adj2 (money or monetary)).ti,ab,kw.
57. Statistical Model/
58. economic model*.ab,kw.
59. Probability/
60. markov.ti,ab,kw.
61. markov.ti,ab,kw.
62. monte carlo method/
63. monte carlo.ti,ab,kw.
64. Decision Theory/
65. Decision Tree/
66. (decision* adj2 (tree* or analy* or model*)).ti,ab,kw.
67. 49 or 50 or 51 or 52 or 53 or 54 or 55 or 56 or 57 or 58 or 59 or 60 or 61 or 62 or 63 or 64 or 65 or 66
68. 25 and 67
69. 33 or 48 or 68

# 4. Web of Science search strategy

**WOS (via THOMSON REUTERS)**

Indexes=SCI-EXPANDED, SSCI, A&HCI, CPCI-S, CPCI-SSH, ESCI Timespan=All years

1. TS=(fatty acid*)
2. TS=(omega 3 or omega 6)
3. TS= ((eicosa*or icosa* or docosahex* or alpha‐linol*) SAME acid*)
4. TS=(PUFA*)
5. TS= (oil* SAME (fish* or flax or linseed))
6. TS =(EFA or EPA or MaxEPA or DHA or ALA)
7. #6 OR #5 OR #4 OR #3 OR #2 OR #1
8. TS=(autis* or asperger* or "pervasive developmental " or (pervasive NEAR/3 child))
9. #8 AND #7
10. TS=(random* or control* or trial* or groups* or effectiveness or evaluation or placebo*)
11. #10 AND #9

# 5. PsycINFO search strategy

**PsycINFO (via OVID) 1806 to 2018 October 26.**

1. Fatty Acids/
2. (omega 3 or omega 6).tw.
3. (eicosa$ or icosa$ or docosahex$).tw.
4. (PUFA or PUFAs).tw.
5. (EFA or EPA or MaxEPA or DHA or ALA).tw.
6. (fish$ oil$ or flax oil$ or linseed oil$).tw.
7. 1 or 2 or 3 or 4 or 5 or 6
8. pervasive developmental disorders.mp. or exp Autism Spectrum Disorders/
9. pervasive development$ disorder$.tw.
10. (pervasive adj3 child$).tw.
11. autis$.tw.
12. asperger$.tw.
13. (autis$ or ASD or ASDs).tw.
14. (ASD or ASDs or PDD or PDDs).tw.
15. Kanner$.tw.
16. 8 or 9 or 10 or 11 or 12 or 13 or 14 or 15
17. 7 and 16

We searched for ongoing clinical trials and unpublished trials via Internet searches on the following web-sites:

- ClinicalTrials.gov ([www.clinicaltrials.gov](http://www.clinicaltrials.gov/));
- World Health Organization (WHO) International Clinical Trials Registry Platform (ICTRP) ([apps.who.int/trialsearch/](http://apps.who.int/trialsearch/)).
- Australian New Zealand Clinical Trial Registry (ANZCTR) (anzctr.org.au/BasicSearch.aspx)

# Search Strategy for equity, acceptability and feasibility.

**MEDLINE search strategy**

**MEDLINE (Ovid MEDLINE® Epub Ahead of Print, In-Process & Other Non-Indexed Citations, Ovid MEDLINE® Daily and Ovid MEDLINE®) 1946 to 2018 October 26.**

("Child Development Disorders, Pervasive" OR (autis* OR ASD OR asds OR PDD OR pdds) OR "pervasive developmental disorder" OR asperg* OR kanner*) AND (child OR adolescent OR pediatrics OR (child OR schoolchild* OR kid OR kids OR toddler* OR adoles* OR teen* OR boy OR girl* OR minors* OR underag* OR under age OR juvenil* OR youth* OR kindergar* OR puberty OR pubescen* OR prepubescen* OR prepuberty* OR pediatric* OR paediatric* OR peadiatric* OR preschool* OR school age)) AND ("Attitude to Health" OR "Patient Participation" OR "Patient Preference" OR (choice OR choices) OR value* OR "health state values" OR valuation* OR expectation* OR attitude* OR acceptab* OR "point of view" OR "patient* participation" OR "user* participation" OR "user* perspective*" OR "patient* perce*" OR "user* perce*" OR "user view*" OR "patient* view*" OR "Feasibility" OR "barriers and facilitators" OR "complementary" OR "attitudes" OR "alternative" OR "prevalence" OR "epidemiology" OR "surveys and questionnaires) AND (" fatty acids "OR " fatty acids, unsaturated "OR " fatty acids, omega-3 "OR " fatty acids, omega-6 "OR " alpha-linolenic acid "OR " docosahexaenoic acids "OR " eicosapentaenoic acid "OR (omega 3 or omega 6) OR " polyunsaturat* fatty acid* "OR PUFA OR " fish oils "OR " linseed oil "OR (EFA OR EPA OR MaxEPA OR DHA OR ALA OR " complementary OR (oil* OR fish* OR flax OR linseed)).
