# Supplementary material for: Equity, acceptability and feasibility of using polyunsaturated fatty acids in children and adolescents with autism spectrum disorder: a rapid systematic review
Source: Health Qual Life Outcomes. 2020 Apr 16;18:101. doi: 10.1186/s12955-020-01354-8 (PMC7164335; doi:10.1186/s12955-020-01354-8)
Supplement: Supplementary file 2 — Additional file 2. References for included and excluded studies. [file 12955_2020_1354_MOESM2_ESM.docx]

**Additional file 2. Full references for included and excluded studies.**

# References for included randomized controlled trials

- Amminger 2007
  - Amminger GP, Berger GE, Schafer MR, Klier C, Friedrich MH, Feucht M. Omega-3 fatty acids supplementation in children with autism: A double-blind randomized, placebo-controlled pilot study. Biological Psychiatry 2007;61:551-3.
- Bent 2011
  - * Bent S, Bertoglio K, Ashwood P, Bostrom A, Hendren RL. A pilot randomized controlled trial of omega-3 fatty acids for autism spectrum disorder. Journal of Autism and Developmental Disorders 2011;41(5):545-54
  - NCT00786799. Omega-3 Fatty Acids for Autism Treatment. clinicaltrial.gov (first posted 6^th^ November 2008).
- Bent 2014
  - * Bent S, Hendren RL, Zandi T, Law K, Choi JE, Widjaja F, Kalb L, Nestle J, Law P. Internet-based, randomized, controlled trial of omega-3 fatty acids for hyperactivity in autism. J Am Acad Child Adolesc Psychiatry 2014;53(6):658-66.
  - NCT01694667. Omega-3 Fatty Acids for Hyperactivity Treatment in Autism Spectrum Disorder. clinicaltrial.gov (first posted 27^th^ September 2012).
- Johnson 2010
  - Johnson CR, Handen BL, Zimmer M, Sacco K. Polyunsaturated fatty acid supplementation in young children with autism. Journal of Developmental and Physical Disabilities 2010;22:1-10.
- Keim 2018
  - Keim SA, Gracious B, Boone KM, Klebanoff MA, Rogers LK, Rausch J, Coury DL, Sheppard KW, Husk J, Rhoda DA. ω-3 and ω-6 Fatty Acid Supplementation May Reduce Autism Symptoms Based on Parent Report in Preterm Toddlers. J Nutr 2018;148(2):227-35.
  - Boone KM, Gracious B, Klebanoff MA, Rogers LK, Rausch J, Coury DL, Keim SA. Corrigendum to "Omega-3 and -6 fatty acid supplementation and sensory processing in toddlers with ASD symptomology born preterm: a randomized controlled trial". Early Human Development 2018.
  - Boone KM, Gracious B, Klebanoff MA, Rogers LK, Rausch J, Coury DL, Keim SA.. Omega-3 and -6 fatty acid supplementation and sensory processing in toddlers with ASD symptomology born preterm: a randomized controlled trial. Early Human Development 2017;115:64-70.
  - Sheppard KW, Boone KM, Gracious B, Klebanoff MA, Rogers LK, Rausch J, BartlettC, Coury DL, Keim SA.. Effect of Omega-3 and -6 Supplementation on Language in Preterm Toddlers Exhibiting Autism Spectrum Disorder Symptoms.. J Autism Dev Disord 2017;47(11):3358-69.
  - NCT01683565. Preemie Tots: A Pilot Study to Understand the Effects of Prematurity in Toddlerhood. clinicaltrial.gov (first posted: 12^th^ September 2012).
- Mankad 2015
  - * Mankad D, Dupuis A, Smile S, Roberts W, Brian J, Lui T, Genore L, Zaghloul D, Iaboni A, Marcon PM, Anagnostou E. A randomized, placebo controlled trial ofomega-3 fatty acids in the treatment of young children with autism. Mol Autism 2015;6:18.
  - NCT01248728. Omega-3 Fatty Acids For Treatment Of Young Children With Autism (OMG). clinicaltrial.gov (first posted 18^th^ May 2016).
- Mazahery 2018
  - * Mazahery H, Conlon CA, Beck KL, Mugridge O, Kruger MC, Stonehouse W, Camargo CA, Meyer BJ, Jones B, von Hurst PR. A randomised controlled trial of vitamin D and omega-3 long chain polyunsaturated fatty acids in the treatment of irritability and hyperactivity among children with Autism Spectrum Disorder. Journal of Steroid Biochemistry and Molecular Biology 2018. DOI: <https://doi.org/10.1016/j.jsbmb.2018.10.017>
  - Mazahery H, Conlon C, Beck KL, Kruger MC, Stonehouse W, Camargo CA Jr, Meyer BJ, Tsang B, Mugridge O, von Hurst PR. Vitamin D and omega-3 fatty acid supplements in children with autism spectrum disorder: a study protocol for a factorial randomised, double-blind, placebo-controlled trial. Trials 2016;17(1):295.
  - ACTRN12615000144516. Effect of Vitamin D and Omega-3 Fatty Acid Supplements on behavioural measures in Children with Autism Spectrum Disorder (ASD): A randomised, double-blind, placebo-controlled trial. anzctr.org.au/Trial (first registered 16^th^ February 2015).
- Parellada 2017
  - Moreno C, Calvo-Escalona R, Gutierrez S, Graell M, Romo J, Dorado ML, Giraldez ML, Llorente C, Arango C, Parellada M. Effect of omega-3 polyunsaturated fatty acids on oxidative stress in children and adolescents with autism spectrum disorders. European neuropsychopharmacology 2014;24:S725.
  - * Parellada M, Llorente C, Calvo R, Gutierrez S, Lázaro L, Graell M, GuisasolaM, Dorado ML, Boada L, Romo J, Dulin E, Sanz I, Arango C, Moreno C. Randomized trial of omega-3 for autism spectrum disorders: Effect on cell membrane composition and behavior. Eur Neuropsychopharmacol 2017;27(12):1319-30.
- Voigt 2014
  - * Voigt RG, Mellon MW, Katusic SK, Weaver AL, Matern D, Mellon B, Jensen CL,Barbaresi WJ. Dietary docosahexaenoic acid supplementation in children with autism. Journal of Pediatric Gastroenterology and Nutrition 2014;58(6):715-22.
  - NCT00577447. Docosahexaenoic Acid in the Treatment of Autism. clinicaltrial.gov (first posted 20^th^ December 2007).
- Yui 2012
  - * Yui K, Koshiba M, Nakamura S, Kobayashi Y. Effects of large doses of arachidonic acid added to docosahexaenoic acid on social impairment in individuals with autism spectrum disorders: a double-blind, placebo-controlled, randomized trial. Journal of clinical psychopharmacology 2012;32(2):200-6.
  - Yui K, Koshiba K, Nakamura S. Effects of adding large doses of arachidonic acid to docosahexaenoic acid on social impairment in individuals with autism spectrum disorders. Current psychopharmacology 2013;2(1):84-90.
  - NCT01154894. Dietary Fatty Acids Improve Social Impairment in Autism Spectrum Disorders (Fatty acid). clinicaltrial.gov (first posted 1^st^ July 2010).

# References for studies excluded from randomized controlled trial selection, with reasons for exclusion

- Adams 2018 (wrong intervention)
  - Adams JB, Audhya T, Geis E, Gehn E, Fimbres V, Pollard EL, Mitchell J, Ingram J, Hellmers R, Laake D, Matthews JS, Li K, Naviaux JC, Naviaux RK, Adams RL,Coleman DM, Quig DW. Comprehensive Nutritional and Dietary Intervention forAutism Spectrum Disorder-A Randomized, Controlled 12-Month Trial. Nutrients 2018;10(3):E369.
  - NCT02059577. Nutritional and Dietary Treatment Study for Children/Adults With Autism. clinicaltrial.gov (first posted 11^th^ Febraury 2014).
- Johnson 2003 (not a randomized controlled trial)
  - Johnson SM, Hollander E. Evidence that eicosapentaenoic acid is effective in treating autism. Journal of Clinical Psychiatry 2003;64:848-9.
- Meguid 2008 (not a randomized controlled trial)
  - Meguid NA, Atta HM, Gouda AS, Khalil RO. Role of polyunsaturated fatty acids in the management of Egyptian children with autism. Clinical Biochemistry 2008;41:1044-8.
- Meiri 2009 (not a randomized controlled trial)
  - Meiri G, Bichovsky Y, Belmaker RH. Omega 3 fatty acid treatment in autism. Journal of Child and Adolescent Psychopharmacology 2009;19(4):449-51.
- NCT01695200 (not a randomized controlled trial)
  - NCT01695200. Omega-3 Fatty Acids in Autism Spectrum Disorders. clinicaltrial.gov (first posted 27^th^ September 2012).
- Ooi 2015 (not a randomized controlled trial)
  - Ooi YP, Weng SJ, Jang LY, Low L, Seah J, Teo S, Ang RP, Lim CG, Liew A, Fung DS, Sung M. Omega-3 fatty acids in the management of autism spectrum disorders: findings from an open-label pilot study in Singapore. Eur J Clin Nutr. 2015 Aug;69(8):969-71. doi: 10.1038/ejcn.2015.28. Epub 2015 Mar 25. PubMed PMID: 25804268.
- Patrick 2005 (not a randomized controlled trial)
  - Patrick L, Salik R. The effect of essential fatty acid supplementation on language development and learning skills in autism and Asperger's syndrome. Autism Asperger's Digest 2005;Jan-Feb:36-7.
- Politi 2008 (not a randomized controlled trial)
  - Politi P, Cena H, Comelli M, Marrone G, Allegri C, Emanuele E, et al. Behavioral effects of omega-3 fatty acid supplementation in young adults with severe autism: an open label study. Archives of Medical Research 2008;39(7):682-5.

**Ongoing studies**

- NCT03550209. Fatty Acid Supplementation in Children With ASD (Omega Heroes). clinicaltrial.gov (first posted 8^th^ June 8 2018).
- NCT03757585. Management of Emotional Dysregulation in Youth With Non-verbal Learning Disability (NVLD) and/or Autism Spectrum Disorders (ASD) Using Telepsychiatry of Complementary and Alternative Treatments. clinicaltrial.gov (first posted 29^th^ November 2018).

**Studies awaiting classification**

- NCT01260961. Developing treatment, treatment validation and treatment scope in the setting of an autism (first posted 15^th^ December 2010).
- NCT01248130. Omega-3 fatty acids monotherapy in children and adolescents with autism spectrum disorders (first posted 25^th^ November 2010).
- NCT00467818. Omega 3 Fatty Acids in the Treatment of Children With Autism Spectrum Disorders (first posted 1^st^ May 2007)
- NCT03620097. Evaluate the Efficacy and Safety of DHA in the Adjuvant Treatment of Children With ASD (first posted 8^th^ August 2018).

# References for studies included in equity, acceptability and feasibility evaluation

- Belmaker 2013
  - Belmaker RH, Meiri G. Omega-3 Fatty Acids in the Treatment of Autism Spectrum Disorder. Comprehensive Guide to Autism. 2013. Publisher: Springer; Patel VB, Preedy VR, Martin CR, Eds.
- Hock 2015
  - Hock R, Kinsman A, Ortaglia A. Examining treatment adherence among parents of children with autism spectrum disorder. Disabil Health J. 2015;8(3):407-13.
- Hopf 2016
  - Hopf KP, Madren E, Santianni KA. Use and Perceived Effectiveness of Complementary and Alternative Medicine to Treat and Manage the Symptoms of Autism in Children: A Survey of Parents in a Community Population. J Altern Complement Med. 2016;22(1):25-32.
- Huang 2013
  - Huang A, Seshadri K, Matthews TA, Ostfeld BM. Parental perspectives on use, benefits, and physician knowledge of complementary and alternative medicine in children with autistic disorder and attention-deficit/hyperactivity disorder. J Altern Complement Med. 2013;19(9):746-50.
- Meiri 2009
  - Meiri G, Bichovsky Y, Belmaker RH. Omega 3 fatty acid treatment in autism. J Child Adolesc Psychopharmacol. 2009;19(4):449-51.
- Nadon 2011
  - Nadon G, Feldman DE, Dunn W, Gisel E. Association of sensory processing and eating problems in children with autism spectrum disorders. Autism Res Treat. 2011;2011:541926
- Ooi 2015
  - Ooi YP, Weng SJ, Jang LY, Low L, Seah J, Teo S, et al. Omega-3 fatty acids in the management of autism spectrum disorders: findings from an open-label pilot study in Singapore. Eur J Clin Nutr. 2015 Aug;69(8):969-71. doi: 10.1038/ejcn.2015.28.
- Press 2011
  - Press, R. The Omega-3 Fatty Acid Composition and Cost Analysis of Fish Oil Supplements: Fishing for the Best Deals. 2011. The Ohio State University. June 2011 Project Advisor: Dr. Martha A. Belury, PhD, R.D. Carol S. Kennedy. Department of Human Nutrition, The Ohio State University, Columbus, OH 43210, USA.
- Salomone 2015
  - Salomone E, Charman T, McConachie H, Warreyn P; Working Group 4, COST Action ‘Enhancing the Scientific Study of Early Autism’. Prevalence and correlates of use of complementary and alternative medicine in children with autism spectrum disorder in Europe. Eur J Pediatr. 2015;174(10):1277-85.
- Watters 2012
  - Watters CA, Edmonds CM Rosner LS Sloss KP Leung PS. A Cost Analysis of EPA and DHA in Fish, Supplements, and Foods. J Nutr Food Sci. 2012;2(8):159.

# References for studies excluded from equity, acceptability and feasibility evaluation, with reasons for exclusion

- Bell 2004 (no outcome of interest)
  - Bell JG, MacKinlay EE, Dick JR, MacDonald DJ, Boyle RM, Glen AC. Essential fatty acids and phospholipase A2 in autistic spectrum disorders. Prostaglandins Leukot Essent Fatty Acids. 2004 Oct;71(4):201-4. PubMed PMID: 15301788
- Fischetti 2012 (wrong intervention)
  - Fischetti AT, Wilder DA, Myers K, Leon-Enriquez Y, Sinn S, Rodriguez R. An evaluation of evidence-based interventions to increase compliance among children with autism. J Appl Behav Anal. 2012 Winter;45(4):859-63. doi:10.1901/jaba.2012.45-859. PubMed PMID: 23322942; PubMed Central PMCID: PMC3545511
- Guerra 2007 (wrong study population)
  - Guerra A, Demmelmair H, Toschke AM, Koletzko B. Three-year tracking of fatty acid composition of plasma phospholipids in healthy children. Ann Nutr Metab. 2007;51(5):433-8. Epub 2007 Nov 20. PubMed PMID: 18025816.
- Harrington 2006 (no outcome of interest)
  - Harrington JW, Rosen L, Garnecho A, Patrick PA. Parental perceptions and use of complementary and alternative medicine practices for children with autistic spectrum disorders in private practice. J Dev Behav Pediatr. 2006 Apr;27(2 Suppl):S156-61. PubMed PMID: 16685182.
- Hock 2012 (wrong intervention)
  - Hock R, Ahmedani BK. Parent perceptions of autism severity: exploring the social ecological context. Disabil Health J. 2012 Oct;5(4):298-304. doi: 10.1016/j.dhjo.2012.06.002. Epub 2012 Aug 2. PubMed PMID: 23021742.
- Hoyos 2008 (wrong study population)
  - Hoyos C, Almqvist C, Garden F, Xuan W, Oddy WH, Marks GB, Webb KL. Effect of omega 3 and omega 6 fatty acid intakes from diet and supplements on plasma fatty acid levels in the first 3 years of life. Asia Pac J Clin Nutr. 2008;17(4):552-7. PubMed PMID: 19114389.
- Ismail 2014 (wrong study population)
  - Ismail A, Rice HB. Considerations for incorporating eicosapentaenoic and docosahexaenoic omega-3 fatty acids into the military food supply chain. Mil Med. 2014 Nov;179(11 Suppl):157-61. doi: 10.7205/MILMED-D-14-00148. Review. PubMed PMID: 25373100.
- James 2011 (no outcome of interest)
  - James S, Montgomery P, Williams K. Omega-3 fatty acids supplementation for autism spectrum disorders (ASD). Cochrane Database Syst Rev. 2011 Nov 9;(11):CD007992. doi: 10.1002/14651858.CD007992.pub2. Review. PubMed PMID: 22071839.
- Meguid 2008 (no outcome of interest)
  - Meguid NA, Atta HM, Gouda AS, Khalil RO. Role of polyunsaturated fatty acids in the management of Egyptian children with autism. Clin Biochem. 2008 Sep;41(13):1044-8. doi: 10.1016/j.clinbiochem.2008.05.013. Epub 2008 Jun 12. PubMed PMID: 18582451
- Politi 2008 (wrong study population)
  - Politi P, Cena H, Comelli M, Marrone G, Allegri C, Emanuele E, Ucelli di Nemi S. Behavioral effects of omega-3 fatty acid supplementation in young adults with severe autism: an open label study. Arch Med Res. 2008 Oct;39(7):682-5. doi: 10.1016/j.arcmed.2008.06.005. PubMed PMID: 18760197
- Sadler 2018 (no outcome of interest)
  - Sadler MJ. A review of economic evaluations for beneficial health outcomes of micronutrient and long-chain omega-3 fatty acid supplementation, International Journal of Food Sciences and Nutrition, 2018; 69:3, 262-282, DOI: 10.1080/09637486.2017.1365825
- Schiff 2011 (case report)
  - Schiff A, Tarbox J, Lanagan T, Farag P. Establishing compliance with liquid medication administration in a child with autism. J Appl Behav Anal. 2011 Summer;44(2):381-5. doi: 10.1901/jaba.2011.44-381. PubMed PMID: 21709797; PubMed Central PMCID: PMC3120077.
- Sheppard 2017 (no outcome of interest)
  - Sheppard KW, Boone KM, Gracious B, Mark A. Klebanoff MA, Rogers LK Bartlett JC, Coury DL, Keim SA. Effect of Omega-3 and -6 Supplementation on Language in Preterm Toddlers Exhibiting Autism Spectrum Disorder Symptoms. J Autism Dev Disord 2017. DOI 10.1007/s10803-017-3249-3
- Vancassel 2001 (no outcome of interest)
  - Vancassel S, Durand G, Barthélémy C, Lejeune B, Martineau J, Guilloteau D, Andrès C, Chalon S. Plasma fatty acid levels in autistic children. Prostaglandins Leukot Essent Fatty Acids. 2001 Jul;65(1):1-7. PubMed PMID: 11487301
