# Supplementary material for: Equity, acceptability and feasibility of using polyunsaturated fatty acids in children and adolescents with autism spectrum disorder: a rapid systematic review
Source: Health Qual Life Outcomes. 2020 Apr 16;18:101. doi: 10.1186/s12955-020-01354-8 (PMC7164335; doi:10.1186/s12955-020-01354-8)
Supplement: Supplementary file 3 — Additional file 3. Quality of Health Economic Studies (QHES) instrument rating for included cost-analysis studies. [file 12955_2020_1354_MOESM3_ESM.docx]

**Additional file 3: quality evaluation of included cost-analysis studies through Quality of Health Economic Studies (QHES) instrument (Ofman 2003).**

|  |  | *Study (author, year)* | |
| --- | --- | --- | --- |
| *Questions* | ***Points*** | ***Press, 2011* [32]** | ***Watters et al., 2012* [33]** |
| 1. Was the study objective presented in a clear, specific, and measurable manner? | 7 | ✔ | ✔ |
| 2. Were the perspective of the analysis (societal, third-party payer, etc.) and reasons for its selection stated? | 4 | ✔ | ✔ |
| 3. Were variable estimates used in the analysis from the best available source (i.e., randomized control trial - best, expert opinion - worst)? | 8 | ✔ | ✔ |
| 4. If estimates came from a subgroup analysis, were the groups prespecified at the beginning of the study? | 1 |  |  |
| 5. Was uncertainty handled by (1) statistical analysis to address random events, (2) sensitivity analysis to cover a range of assumptions? | 9 |  |  |
| 6. Was incremental analysis performed between alternatives for resources and costs? | 6 |  |  |
| 7. Was the methodology for data abstraction (including the value of health states and other benefits) stated? | 5 | ✔ | ✔ |
| 8. Did the analytic horizon allow time for all relevant and important outcomes? Were benefits and costs that went beyond 1 year discounted (3% to 5%) and justification given for the discount rate? | 7 |  |  |
| 9. Was the measurement of costs appropriate and the methodology for the estimation of quantities and unit costs clearly described? | 8 | ✔ | ✔ |
| 10. Were the primary outcome measure(s) for the economic evaluation clearly stated and did they include the major short-term, long-term, and negative outcomes? | 6 | ✔ | ✔ |
| 11. Were the health outcomes measures/scales valid and reliable? If previously tested valid and reliable measures were not available, was justification given for the measures/scales used? | 7 |  |  |
| 12. Were the economic model (including structure), study methods and analysis, and the components of the numerator and denominator displayed in a clear, transparent manner? | 8 | ✔ | ✔ |
| 13. Were the choice of economic model, main assumptions, and limitations of the study stated and justified? | 7 | ✔ | ✔ |
| 14. Did the author(s) explicitly discuss direction and magnitude of potential biases? | 6 | ✔ |  |
| 15. Were the conclusions/recommendations of the study justified and based on the study results? | 8 | ✔ | ✔ |
| 16. Was there a statement disclosing the source of funding for the study? | 3 |  |  |
| TOTAL POINTS | 100 | **67** | **61** |
