# Supplementary material for: Equity, acceptability and feasibility of using polyunsaturated fatty acids in children and adolescents with autism spectrum disorder: a rapid systematic review
Source: Health Qual Life Outcomes. 2020 Apr 16;18:101. doi: 10.1186/s12955-020-01354-8 (PMC7164335; doi:10.1186/s12955-020-01354-8)
Supplement: Supplementary file 4 — Additional file 4. Newcastle-Ottawa Scale for included cross-sectional studies. [file 12955_2020_1354_MOESM4_ESM.doc]

| Study | Representativeness of the sample | Sample size | Non-respondents | Ascertainment of the exposure (risk factor) (maximum 2 stars) | Comparability of outcome groups (maximum 2 stars) | Assessment of the outcome (maximum 2 stars) | Statistical test | Total stars (maximum 10 stars) |
| --- | --- | --- | --- | --- | --- | --- | --- | --- |
| *Hock et al., 2015* | * | * |  | ** | * | * | * | 7 |
| *Hopf et al., 2016* | * | * |  | * | ** | * |  | 6 |
| *Huang et a., 2013* |  | * |  |  | * | * | * | 4 |
| *Nadon et al., 2011* | * | * | * | ** | * | * | * | 8 |
| *Salomone et al., 2015* | * | * |  | ** | * | * | * | 7 |

**Additional file 4. Quality assessment of cross-sectional included studies through Newcastle - Ottawa Quality Assessment Scale (adapted for cross sectional studies) (Modesti 2016)**
