# Supplementary material for: Equity, acceptability and feasibility of using polyunsaturated fatty acids in children and adolescents with autism spectrum disorder: a rapid systematic review
Source: Health Qual Life Outcomes. 2020 Apr 16;18:101. doi: 10.1186/s12955-020-01354-8 (PMC7164335; doi:10.1186/s12955-020-01354-8)
Supplement: Supplementary file 5 — Additional file 5. ROBINS-I for included before-after studies. [file 12955_2020_1354_MOESM5_ESM.docx]

| Study | Confounding | Selection bias | Measurement of interventions | Departures from intended interventions | Missing data | Measurement of outcomes | Selection of reported results | Overall risk of bias |
| --- | --- | --- | --- | --- | --- | --- | --- | --- |
| *Belmaker et al., 2013* | Serious risk | Moderate risk | Serious risk | Critical risk | Serious risk | Serious risk | No information | Critical risk |
| *Meiri et al., 2009* | Serious risk | No information | Low risk | Moderate risk | Low risk | Serious risk | Serious risk | Serious risk |
| *Ooi et al., 2015* | Serious risk | Moderate risk | Low risk | No information | No information | Moderate risk | Low risk | Serious risk |

**Additional file 5. Risk of bias of included before-after studies, for the outcome “adherence”, as assessed through Risk Of Bias In Non-randomized Studies - of Interventions (ROBINS-I) tool**
