# Supplementary material for: Equity, acceptability and feasibility of using polyunsaturated fatty acids in children and adolescents with autism spectrum disorder: a rapid systematic review
Source: Health Qual Life Outcomes. 2020 Apr 16;18:101. doi: 10.1186/s12955-020-01354-8 (PMC7164335; doi:10.1186/s12955-020-01354-8)
Supplement: Supplementary file 6 — Additional file 6. Risk of bias summary for included randomized controlled trials. [file 12955_2020_1354_MOESM6_ESM.docx]

**Additional file 6. Risk of Bias Summary for randomized controlled trials**

**These are review authors' judgments about each risk of bias item for each included study.**

| **Study** | **Random sequence generation**  **(selection bias)** | **Allocation concealment (selection bias)** | **Blinding (performance bias and detection bias)** | **Incomplete outcome data**  **(attrition bias)** | **Selective reporting (reporting bias)** | **Other bias** |
| --- | --- | --- | --- | --- | --- | --- |
| Amminger, 2007 ^1^ | 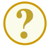 | 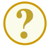 | 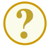 | 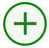 | 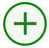 | 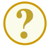 |
| Bent, 2011 ^2^ | 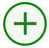 | 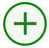 | 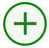 | 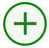 | 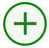 | 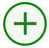 |
| Bent, 2014 ^3^ | 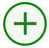 | 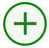 | 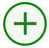 | 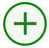 | 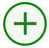 | 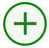 |
| Johnson, 2010 ^4^ | **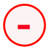** | 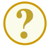 | **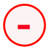** | 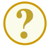 | 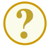 | 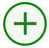 |
| Keim, 2018 ^5^ | 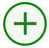 | 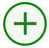 | 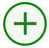 | 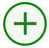 | 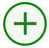 | 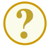 |
| Mankad, 2015 ^6^ | 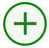 | 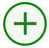 | 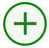 | 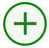 | 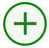 | 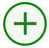 |
| Mazahery, 2018 ^7^ | 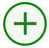 | 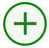 | 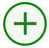 | 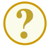 | 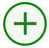 | 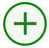 |
| Parellada, 2017 ^8^ | 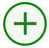 | 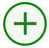 | 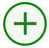 | 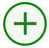 | 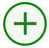 | 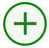 |
| Voigt, 2014 ^9^ | 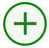 | 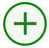 | 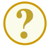 | **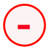** | 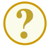 | 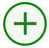 |
| Yui, 2012 ^10^ | 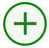 | 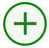 | 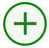 | 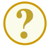 | 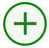 | 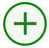 |

Abbreviations:
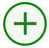
 = Low risk of bias;
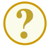
 = Unclear risk of bias; **
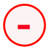
** = High risk of bias.

**References**

1. Amminger GP, Berger GE, Schafer MR, Klier C, Friedrich MH, Feucht M. Omega-3 fatty acids supplementation in children with autism: A double-blind randomized, placebo-controlled pilot study. Biological Psychiatry 2007;61:551-3. doi: 10.1016/j.biopsych.2006.05.007
2. Bent S, Bertoglio K, Ashwood P, Bostrom A, Hendren RL. A pilot randomized controlled trial of omega-3 fatty acids for autism spectrum disorder. Journal of Autism and Developmental Disorders 2011;41(5):545-54. doi: 10.1007/s10803-010-1078-8
3. Bent S, Hendren RL, Zandi T, Law K, Choi JE, Widjaja F, Kalb L, Nestle J, Law P. Internet-based, randomized, controlled trial of omega-3 fatty acids for hyperactivity in autism. J Am Acad Child Adolesc Psychiatry 2014;53(6):658-66. doi: 10.1016/j.jaac.2014.01.018
4. Johnson CR, Handen BL, Zimmer M, Sacco K. Polyunsaturated fatty acid supplementation in young children with autism. Journal of Developmental and Physical Disabilities 2010;22:1-10. doi: 10.1016/j.arcmed.2008.06.005
5. Keim SA, Gracious B, Boone KM, Klebanoff MA, Rogers LK, Rausch J, Coury DL, Sheppard KW, Husk J, Rhoda DA. ω-3 and ω-6 Fatty Acid Supplementation May Reduce Autism Symptoms Based on Parent Report in Preterm Toddlers. J Nutr. 2018 Feb 1;148(2):227-235. doi: 10.1093/jn/nxx047.
6. Mankad D, Dupuis A, Smile S, Roberts W, Brian J, Lui T, Genore L, Zaghloul D, Iaboni A, Marcon PM, Anagnostou E. A randomized, placebo controlled trial ofomega-3 fatty acids in the treatment of young children with autism. Mol Autism 2015;6:18. doi: 10.1186/s13229-015-0010-7
7. Mazahery H, Conlon CA, Beck KL, Mugridge O, Kruger MC, Stonehouse W, Camargo CA, Meyer BJ, Jones B, von Hurst PR. A randomised controlled trial of vitamin D and omega-3 long chain polyunsaturated fatty acids in the treatment of irritability and hyperactivity among children with Autism Spectrum Disorder. Journal of Steroid Biochemistry and Molecular Biology 2018. doi: 10.1016/j.jsbmb.2018.10.017
8. Parellada M, Llorente C, Calvo R, Gutierrez S, Lázaro L, Graell M, GuisasolaM, Dorado ML, Boada L, Romo J, Dulin E, Sanz I, Arango C, Moreno C. Randomizedtrial of omega-3 for autism spectrum disorders: Effect on cell membrane composition and behavior. Eur Neuropsychopharmacol 2017;27(12):1319-30. doi: 10.1016/j.euroneuro.2017.08.426
9. Voigt RG, Mellon MW, Katusic SK, Weaver AL, Matern D, Mellon B, Jensen CL,Barbaresi WJ. Dietary docosahexaenoic acid supplementation in children with autism. Journal of Pediatric Gastroenterology and Nutrition 2014;58(6):715-22. doi: 10.1097/MPG.0000000000000260
10. Yui K, Koshiba M, Nakamura S, Kobayashi Y. Effects of large doses of arachidonic acid added to docosahexaenoic acid on social impairment in individuals with autism spectrum disorders: a double-blind, placebo-controlled, randomized trial. Journal of clinical psychopharmacology 2012;32(2):200-6. doi: 10.1097/JCP.0b013e3182485791
